# Biochemical and molecular responses of *Spodoptera frugiperda* to insecticide exposure: detoxification enzymes, gene expression, and genotoxic effects

**DOI:** 10.1038/s41598-026-45372-w

**Published:** 2026-04-20

**Authors:** Ramy E. El-Ansary, Kreema A. El-Lebody, Raed A. Aburawash, Shaymaa H. Mahmoud, Ghada M. El-Sayed, Mai I. Hussein

**Affiliations:** 1 Zoology and Entomology Department, Faculty of Science, Al-Azhar University, Cairo, Egypt; 2 Plant Protection Institute, Agriculture Research Centre Dokki, Giza, 12627 Egypt; 3 Department of Livestock Research, Arid Lands Cultivation Research Institute, City for Scientifc Research and Technology Applications, New Borg El-Arab, Alexandria, 21934 Egypt; 4 Zoology Department, Faculty of Science, Menoufia University, Shebin El-Kom, Egypt; 5 Microbial Genetics Department, Biotechnology Research Institute, National Research Centre, 33 El-Bohouth St., Dokki, Cairo, 12622 Egypt; 6 Department of Zoology, Faculty of Science, Suez University, Suez, Egypt

**Keywords:** *Spodoptera frugiperda*, Insecticides, Resistance coefficient, Detoxification enzymes, DNA damage, Gene expression, In silico analysis, Biochemistry, Environmental sciences, Molecular biology, Zoology

## Abstract

*Spodoptera frugiperda* (fall armyworm) is a highly destructive agricultural pest with a remarkable ability to develop resistance to insecticides, posing a severe threat to global food security. Understanding the physiological and molecular responses of this species to insecticide exposure is crucial for improving resistance monitoring and developing effective management strategies. In this study, the toxicity and the associated biochemical, molecular, and genotoxic responses of third-instar *S. frugiperda* larvae were investigated following exposure to four commonly used insecticides: emamectin benzoate, indoxacarb, methomyl, and a binary mixture of acetamiprid + bifenthrin. Toxicity bioassays showed that emamectin benzoate was the most potent insecticide (LC_50_ = 0.188 ppm/L), whereas the binary mixture exhibited the highest resistance coefficient (RC = 12.86) relative to the recommended field dose. Biochemical analyses conducted over a seven-day exposure period, significant metabolic alterations were observed. Reduced acetylcholinesterase (AChE) and carboxylesterase (CarE) activities suggested neurotoxic stress, Whereas elevated glutathione S-transferase (GST) and peroxidase (POD) activities reflected enhanced detoxification and oxidative stress responses. Gene expression analyses revealed significant upregulation of SFCYP1, SFCYP2, SFCYP4, SFCYP5, SFRYR, and EF1α, while SFCYP3 was consistently downregulated, indicating activation of detoxification and stress-related pathways. Genotoxic effects were assessed using the comet assay, which demonstrated significant DNAdamage in hemocytes of treated larvae. This damage was characterized by increased tail length, tail DNApercentage, and tail moment, particularly in individuals exposed to emamectin benzoate. Collectively, these findings indicate that sublethal insecticides exposure not only disrupts enzymatic homeostasis but also induces genotoxic stress. These findings demonstrate that *S. frugiperda* exhibits complex adaptive physiological and molecular responses that may contribute to resistance development under sustained insecticide selection pressure. Overall, the results provide integrated biochemical and molecular insights that can support future investigation into insecticide response mechanisms and improve resistance monitoring strategies for this economically important pest.

**Supplementary Information:**

The online version contains supplementary material available at 10.1038/s41598-026-45372-w.

## Introduction

Fall armyworm, *Spodoptera frugiperda* is a highly destructive agricultural pest with a remarkable ability to adapt to various environmental conditions and develop resistance to insecticides, posing a severe threat to global food security^1^. Native to the Americas, *S. frugiperda* has rapidly expanded its geographical range, invading Africa, Asia, and Australia within the last decade^2,3^. Recent studies have documented the presence and genetic diversity of *Spodoptera frugiperda* populations in Egypt, highlighting its rapid spread and adaptation to local agroecosystems^4^.This pest primarily targets maize (Zea mays), but its polyphagous nature allows it to infest over 80 plant species, including rice, sorghum, and cotton, leading to extensive economic losses^5^. Extensive use of chemical insecticides, although effective in the short term, can induce sublethal physiological and biochemical changes in exposed populations, which may serve as early indicators of stress adaptation or tolerance mechanisms rather than confirmed resistance^6,7^.

Several alternative strategies, including biological control approaches, have been explored to reduce reliance on chemical insecticides for managing *Spodoptera frugiperda* infestations^8^ However, chemical insecticides remain the primary method for controlling *S. frugiperda* infestations conducted.

Acute and sublethal exposures to insecticides have been shown to alter the activity of key detoxification enzymes, including cytochrome P450 monooxygenases (P450s), glutathione S-transferases (GSTs), and acetylcholinesterase (AChE). Such enzymatic modulation may represent an adaptive response to chemical stress, helping insects temporarily either metabolize and detoxify insecticides before they reach their target sites, diminishing their efficacy^9^ or cope with toxicants^10^. leading eventually to metabolic resistance involving the upregulation of detoxifying enzyme families such^11^. The pest has demonstrated a high propensity to develop resistance against commonly used insecticides, including pyrethroids, organophosphates, carbamates, and diamide-based compounds^11,12^. Reports indicate significant resistance development in field populations, leading to control failures and increased pesticide application, which exacerbates environmental and human health concerns^13^.

The molecular basis of resistance is further supported by transcriptomic and genomic studies, which reveal differential gene expression patterns associated with insecticide exposure. Cytochrome P450 enzymes are considered key components of insect detoxification systems and are frequently associated with insecticide tolerance mechanisms^7^.

Overexpression of detoxification-related genes, including *CYP* genes, *GSTs*, and *CarEs*, has been reported in resistant *S. frugiperda* populations^14^. In addition to enzymatic detoxification, oxidative stress response mechanisms involving antioxidant enzymes, such as peroxidases and superoxide dismutase (SOD), contribute to resistance adaptation^15^.

Genotoxic stress, including DNA damage, may also occur following insecticide exposure, often mediated by oxidative imbalance^16^.Comet assay is considered as one of the most important tests for genotoxicity determination of insects and fish after exposure to water pollutants, either in the environment or under experimental laboratory treatments^17^. It considered as a rapid, sensitive, and inexpensive method to investigate DNA strand breaks in individual eukaryotic cells^18^.

In addition to experimental assays, in silico docking can provide preliminary insights into potential interactions between insecticides and key molecular targets^8,16,19^. Such computational predictions are informative and considered supportive evidence.

Given the increasing concern about insecticide tolerance in *S. frugiperda*, this study aims to investigate the short-term biochemical and molecular responses of larvae to selected insecticides. The study evaluates acute effects by determining LC50 values and examining the activity of key detoxification enzymes, including P450s, GSTs, CarEs, and AChE, along with oxidative stress markers. In addition, transcriptional responses of resistance- and stress-associated genes (CYP450s, RyR, ace-1, vgsc, and other stress-response genes) will be assessed to elucidate molecular adaptations. DNA damage will be measured using the comet assay to provide insights into genotoxic stress. Overall, the study seeks to characterize adaptive physiological and molecular responses to insecticide exposure, providing baseline information to support future monitoring and integrated pest management strategies, without implying confirmed long-term resistance evolution.

## Materials and methods

### Identification of FAW

#### Morphological identification

Collected insects from infested maize plants were transferred to insectary, Zoology and Entomology Department, Faculty of Science, Al-Azhar University, Egypt. Identification of the larvae and adults performed based on their morphological characteristics according to Passoa^20^, and CABI^21,22^.

#### Molecular identification

To validate species identity, DNA extraction was performed from larvae of FAW using QiAmp mini DNA extraction kit (QIAGEN). Following standard protocols, the mitochondrial cytochrome oxidase subunit I (COI) encoding gene was amplified using primer sets of LCO1490 forward primer 5’-GGT CAA CAA ATC ATA AAG ATA TTG G-3′, and HCO2198 reverse primer 5’-TAA ACT TCA GGG TGA CCA AAA AAT CA-3’ ^23^. Amplified products underwent purification using QIAquick PCR purification kit, both orientations of sequencing. Resulting sequences (670 bp) were added to the NCBI GenBank and subjected to BLAST homology test.

### Insect

A laboratory colony of *S. frugiperda* colony was established from larvae collected from 60-day-old maize plants (hybrid type 324, tripartite) in Beheira Governorate, Egypt (31.0728° N, 30.3063° E). The collected larvae were subsequently reared and maintained in an insectary under controlled laboratory conditions.

### Insect rearing

A laboratory colony of *S. frugiperda *was maintained for four generations under insecticide-free conditions at 27 ± 1 °C, 65 ± 5% relative humidity, and a photoperiod of 16 h light: 8 h dark. Newly hatched larvae were initially reared in 1-L glass jars and fed fresh maize leaves until reaching the third instar. To minimize cannibalism, larvae were subsequently transferred to larger 2-L glass jars containing a thin layer of fine sawdust at the bottom. Standard rearing procedures were followed throughout larval development. Adult moths were maintained in 3-L glass jars and provided with cotton wool soaked in a 10% sucrose solution, which was replaced daily to prevent fungal contamination^24^.

### Insecticides

The tested insecticides were categorized according to the Insecticides Resistance Action Committee (IRAC) mode-of-action classification system (version 11.2, August 2024) and are presented in Table 1.

**Table 1:** Insecticide treatments active ingredients and mode of action.

Insecticides	Active ingredient	Mode of action
Speedo 5.7% (WG) 80 gm/F	Emamectin benzoate	Glutamate-gated chloride channel (Gluc Cl) allostreric modulators
Easo 30% (WG) 60gm/F	Indoxacarb	Voltage-dependent
		Sodium channel blockers
Goldben 90% (SP) 300 gm/F	Methomyl	Acetyl cholinesterase
		(ACh E) inhibitors
Rubek 50% (WP) 100 gm/F	22.7Acetampirid	Nicotinic ACh receptor (nAChR) Compatitive modulators
	27.3 Benfenthrin	Sodium channel modulators

### Bioassays

The toxicity of Indoxacarb, Emamectin benzoate, Methomyl and a binary mixture of 22.7% Acetamiprid + 27.3% Bifenthrin against third instar of *S frugiperda* larvae was evaluated using the maize leaf dipping method^25^, with minor modifications. Briefly, six concentrations were prepared for each insecticide, while distilled water served as the control group. Maize leaves were first washed with water, then dipped in the respective insecticide solutions for 30 s and allowed to air-dry. After drying, the treated leaves were placed in0.25 L glass jars. Ten larvae were introduced into each jar with three replicates per treatment and allowed to feed on the treated leaves for 24 h. Subsequently, larvae were provided with untreated leaves, and the mortality% was recorded three days (72 h) post-treatment^26^. These data were used to estimate lethal and sub-lethal concentrations for each insecticide. The entire bioassay was conducted twice.

### Resistance of *S. frugiperda* to tested insecticides

The Resistance Coefficient (RC) of *S frugiperda* larvae to each insecticide was calculated according to^27^. Lethal concentrations (LC_50_) of Indoxacarb, Emamectin benzoate, Methomyl and a binary mixture of 22.7% Acetamiprid + 27.3% Bifenthrin were applied to third instar larvae as described above. Surviving larvae were subsequently used to evaluate the effects of each insecticide on larval biochemical parameters and the expression of resistance-related genes through qPCR investigation. At three- and seven-days post-exposure, surviving larvae were transferred to sterile 2 ml Eppendorf tubes and immediately stored at -80 °C for subsequent analysis.

### Biochemical assessment

#### Total protein estimation

Third-instar *S. frugiperda* larvae were treated with selected insecticides and homogenized under chilled conditions at 3 and 7 days post-treatment. The homogenates were centrifuged, and the resulting supernatants were stored at − 20 °C for subsequent enzymatic analysis^28^. Total protein content was determined using the Bradford method (Bradford, 1976). Briefly, 50 µL of each sample or bovine serum albumin (BSA) standard (10–100 µg) was mixed with Coomassie Brilliant Blue G-250 dye reagent. The volume was adjusted to 1 mL with 0.1 M phosphate buffer (pH 6.6), followed by the addition of 5 mL of dye reagent. Absorbance was measured at 595 nm within 1 h.

#### Acetylcholinesterase (AChE) determination

AChE activity was determined following the method of Simpson, et al.^29^. The reaction mixture consisted of the enzyme extract, 0.067 M phosphate buffer (pH 7.0), and 3 mM acetylthiocholine bromide (AchBr). After incubation at 37 °C for 30 min, the reaction was terminated by adding alkaline hydroxylamine followed by HCl, and FeCl_3_ was subsequently added. Absorbance was measured at 515 nm to quantify enzyme activity.

#### Phenoloxidase (PO) activity determination

Phenoloxidase (PO) activity was measured following^30^. The reaction mixture contained 2% catechol in 0.1 M phosphate buffer (pH 7) and was incubated at 25 °C for 5 min. Absorbance was recorded at 405 nm to quantify enzyme activity**.**

#### Determination of key metabolic and detoxification enzymes

Amylase activity was determined followingr Ishaaya and Swirski^31^**,** using 1% soluble starch in acetate buffer (pH 5.0) as the substrate. After incubation at 30 °C for 10 min, the reaction was stopped with DNS reagent and boiled. Absorbance was measured at 550 nm. Non-Specific Esterases (α- and β-esterase) were assayed according to Van Asperen^32^ using α- or β-naphthyl acetate as substrates. Following 15 min incubation at 27 °C, diazoblue reagent was added, and absorbance was read at 600 nm (α-naphthol) or 555 nm (β-naphthol). Carboxylesterase (CarE) activity was measured as described by Simpson et al.^29^**,** using (4 mM) methyl paraoxon (MeB) as substrate. The reaction mixture was incubated at 37 °C for 30 min, followed by sequential addition of alkaline hydroxylamine, HCl, and FeCl₃. Absorbance was measured at 515 nm. Lipase activity was determined using a commercial Spectrum Diagnostics kit, based on the hydrolysis of the DGMRE substrate and detection of methylresorufin at 578 nm.

#### Determination of antioxidant enzyme activities and total antioxidant capacity

Glutathione S-Transferase GST activity was measured following Habig et al.^33^. The reaction mixture contained phosphate buffer (pH 6.5), 5 mM reduced glutathione (GSH), enzyme extract, and 1 mM 1-chloro-2,4-dinitrobenzene (CDNB). Absorbance was measured at 340 nm. Peroxidase (POD) activity was assayed according to Hammerschmidt et al.^34^, using 0.05 M pyrogallol and 1% hydrogen peroxide. Absorbance was measured at 420 nm. Superoxide Dismutase (SOD) activity was determined following Nishikimi et al.^35^, based on the inhibition of nitroblue tetrazolium reduction, with absorbance measured at 560 nm. Total Antioxidant Capacity (TAC) was assessed using the phosphomolybdenum method^36^**.** The reaction mixture was incubated at 90 °C for 90 min and absorbance was measured at 695 nm.

### Molecular assessment

#### Gene expression analysis by RT-qPCR

To assess the effects of different insecticide formulations (Easo, Speedo, Rubek, and Goldben) on third-instar larvae, the expression levels of seven defense-related genes (SFCYP1, SFCYP2, SFCYP3, SFCYP4, SFCYP5, SFRYR, and EF1α) were evaluated after 7 days of exposure. Primer sequences for allgenes are provided in Table 2**.** Total RNA was extracted using the RNeasy Mini Kit (Qiagen, Germany), and RNA concentration and purity were determined with a Nanodrop 2000 spectrophotometer (Thermo Scientific, USA). First-strand cDNA was synthesized in a 20 µL reaction using oligo(dT) primers and reverse transcriptase (New England Biolabs) under the following conditions: 42 °C for 1 h and 95 °C for 5 min.

**Table 2:** Oligonucleotide sequences of the primers used in RT-qPCR study.

Primer	Sequence
SFCYP1	F- 5′ GAGCTTACTTCGGCACGTTG 3′
	R- 5′ CAACACTTTCGAGCGGTCG 3′
SFCYP2	F- 5′ GAAGCGTGGCGTAAAGTTCT 3′
	R- 5′ AAGAGCGCAGGTGTTAGGAC 3′
SFCYP3	F- 5′ GAGAAAGTATCCGCCGGGTT 3′
	R- 5′ ACAAGCTCTCCATTCCGATCC 3′
SFCYP4	F- 5′ AAGAACCCTTGGAAACCG 3′
	R- 5′ ATGGGAACACTAAGTCGGGG 3′
SFCYP5	F- 5′ TGTTATCCAAGCAAATTGATCGAG 3′
	R- 5′ GTCAAATGCGGCTGATGACG 3′
SFRYR	F- 5′ CACAGGTGGATCTCTCCCAG 3′
	R- 5′ GCGTCCAACGTAGACACCTT 3′
EF1α	F- 5′ TGGGCGTCAACAAAATGGA 3′
	R- 5′ TCTCCGTGCCAGCCAGAAAT 3′
ß actin	F- 5′ GTGGGCCGCTCTAGGCACCAA 3′
	R- 5′ CTCTTTGATGTCACGCACGATTTC 3′

Quantitative real-time PCR (RT-qPCR) was performed using SYBR Green Master Mix (Fermentas, USA) on a Rotor-Gene 6000 system (QIAGEN). Each 25 µL reaction contained forward and reverse primers (1.5 µL each, 10 pmol/µL), 1 µL of cDNA template (50 ng), 12.5 µL SYBR Green, and nuclease-free water. The cycling conditions included an initial denaturation at 95 °C for 10 min, followed by 40 cycles of denaturation at 95 °C for 15 s, annealing at 60 °C for 30 s, and extension at 72 °C for 30 s. Melting curve analysis confirmed the specificity of amplification. Primer efficiencies were determined using five-fold serial dilutions of cDNA. All primer pairs exhibited efficiencies between 92–105% with correlation coefficients (R^2^) greater than 0.99. β-actin was used as the internal reference gene for normalization, and its expression remained stable across treatments. Relative gene expression was calculated using the 2^ − ΔΔCt method^37^.

#### Genotoxicity analysis by Comet assay (Single cell gel electrophoresis)

DNA damage in hemocytes was assessed using the alkaline comet assay following Singh et al.^38^ with slight modifications. Microscope slides were first coated with 1% normal melting agarose (NMA) and overlaid with a mixture of 0.7% low melting agarose (LMA) and the hemocyte suspension. After gelling, slides were lysed for 1 h at 4 °C in a high-salt lysis buffer to remove membranes and histones. DNA was then unwound in alkaline buffer (pH > 13) for 20 min, followed by electrophoresis at 25 V and 300 mA for 20 min at 4 °C. Slides were then neutralized, dehydrated, and stained with ethidium bromide. Fluorescent nuclei were visualized using an Olympus BX51 microscope, and 100 nuclei per treatment were analyzed using Comet Assay IV software. The following parameters were measured: tail length, % DNA in tail, and tail moment. Together, these metrics reflect the extent of DNA strand breaks in larval hemocytes.

### Statistical analysis

All experiments were conducted with 3–4 biological replicates. Data were first tested for normality using the Shapiro–Wilk test and for homogeneity of variances using Levene’s test to ensure that parametric assumptions were met. For insecticide toxicity, lethal concentration values (LC_50_ and LC_95_), 95% confidence limits (95% CL), regression slopes, and equations were determined using the Ldp-line online tool (https://www.drnabil.22web.org). LC_95_ values were calculated according to Westgard and Westgard^39^, and the toxicity index (TI) at LC_50_ was calculated following^40^. For biochemical, enzymatic and gene expression data, differences among multiple treatment groups were analyzed using one-way ANOVA. When ANOVA indicated significant differences, Duncan’s multiple range (DMR) post hoc test was applied to identify pairwise differences.Independent sample *t*-tests were used to compare treated versus control groups when appropriate. For the comet assay, 100 nuclei per treatment were analyzed, and DNA damage parameters (tail length, % DNA in tail, and tail moment) were compared using ANOVA followed by DMR. All analysis was conducted using SPSS software (version 22.0; IBM Corp., Armonk, NY, USA). Results are presented as mean ± standard error (SE). Statistical significance was set at p < 0.05 graphical representations of data, including error bars and significance indicators, were generated using graphPad Prism 9 (GraphPad Software, San Diego, CA, USA) to facilitate visualization of group differences^39^.

### In silico analysis

#### Homology modelling of protein receptors

Due to the lack of experimentally resolved structures (PDB IDs) for the target protein receptors—glutamate-gated chloride channel (UniProt ID: A0A9R0ER6), sodium channel (NCBI Reference Sequence: XM_050706481.1), and acetylcholinesterase (NCBI Reference Sequence: XM_035573808.2)—their amino acid sequences were analyzed using BLASTp against the Protein Data Bank to identify suitable structural templates. Templates with sequence identity greater than 30% were selected for homology modelling using MODELLER 10.6^41^ based on sequence alignments with homologous proteins. The quality of the generated models was assessed using TM-score, RMSD, and DOPE profiles. Additional validation was performed using the VERIFY3D^42^ and PROCHECK^43^ servers to confirm the structural accuracy and reliability of the predicted models.

#### Molecular docking analysis

Computational analysis were performed to characterize the molecular interactions between acetylcholinesterase, sodium channel protein, and glutamate-gated chloride channel receptors identified as key targets with predicted affinity for the applied insecticides. The 2D structure of Emamectin benzoate was obtained from the PubChem database (CID: 11549937). Active-site residues were predicted using the COACH server^44^, which integrates TM-SITE and S-SITE for complementary binding-site identification.

Hydrogen atoms were added to the receptor models using AutoDock Vina’s MG Tools . The ligand was converted to MOL2 format using Open Babel^45^ and subsequently to PDBQT format with AutoDock Tools. Docking simulations were conducted using grid boxes centered on the predicted binding sites along the x, y, and z axes. Ten docking conformations were generated, and the lowest-energy pose was selected for analysis.

Receptor–ligand interactions, including ligand orientation, hydrogen bonds, and key contact residues, were visualized using BIOVIA Discovery Studio 2021 Client (Dassault Systèmes). All docking experiments were executed using AutoDock Vina 1.2.0 as described by Eberhardt et al.^46^.

## Results

### Molecular identification

Molecular characterization of the FAW larvae was performed using the mitochondrial cytochrome oxidase subunit-I (COI) encoding gene. The nucleotide sequence obtained was compared against reference sequences in the National Center for Biotechnology Information (NCBI) database using the BLAST online tool to determine sequence homology. The CO-I sequence generated in this study were deposited in GenBank under accessions number (PP658132.1).

### Toxicity and resistance of tested insecticides against third-instar *S. frugiperda*

The LC₅₀ values (PPM/L) of the tested insecticides against third-instar *S. frugiperda* larvae after three days of exposure are presented in Table 3, along with their 95% confidence limits (95% CL). Emamectin benzoate exhibited the highest toxicity, with the lowest LC_50_ value of 0.188 PPM/L (95% CL: 0.109–0.324). In contrast, the binary mixture of acetamiprid + bifenthrin showed the lowest toxicity, with the highest LC₅₀ value of 67.261 PPM/L (95% CL: 5.918–764.42). Indoxacarb and methomyl exhibited intermediate toxicities, with LC₅₀ values of 3.592 PPM/L (95% CL: 0.844–15.290) and 5.249 PPM/L (95% CL: 0.679–40.547), respectively.

**Table 3:** Susceptibility and resistance level of *S. frugiperda* to insecticide treatments.

Insecticide	Lc50 (PPM/L)	95%CL		Slope ± SE	Toxicity index %	Lc_95_ (PPM/L)	Recommended dose (PPM/L)	Resistance coefficient (RC)	Resistance level (RL)
		Lower	Upper						
Speedo 5.7% (WG) 80gm/F	0.188	0.109	0.324	0.95 ± 0.09	100	10.13	45.6	0.222	Non
Easo 30% (w G.) 60gm/F	3.592	0.844	15.29	2.25 ± 1.53	5.24	19.23	180	0.106	Non
Goldben 90% (SP) 300gm/F	5.249	0.679	40.547	2.29 ± 1.88	3.58	27.14	2700	0.1	Non
Rubek 50% (WP) 100gm/F	67.261	5.918	764.42	0.82 ± 62	0.28	6430.36	500	12.86	High

Resistance coefficient (RC) = LC_95_ (PPM/L) / Recommended dose (PPM/ L). Where , RC = ≤ 1 No resistance; RC = 1.1—2 Low resistance; RC = 2.1 – 5 Medium resistance ; RC = 5.1—10 High resistance; RC > 10 Very high resistance^27^,.

Based on 95% CL comparisons, emamectin benzoate was significantly more effective than the other tested insecticides. The descending order of treatment efficacy, as determined by the toxicity index (TI %) at LC₅₀, was: emamectin benzoate (100%) > indoxacarb (5.24%) > methomyl (3.58%) > binary acetamiprid + bifenthrin (0.28%).

These findings were further supported by the calculated resistance coefficients (RC). Larvae were highly susceptible to emamectin benzoate, indoxacarb, and methomyl, with RC values of 0.2, 0.106, and 0.1, respectively. In contrast, *S. frugiperda* exhibited a high level of resistance to the binary acetamiprid + bifenthrin treatment, with an RC exceeding ten (12.86), highlighting a marked reduction in sensitivity to this combination.

### Enzymatic activity responses to insecticide treatments

**Fig. 1:**
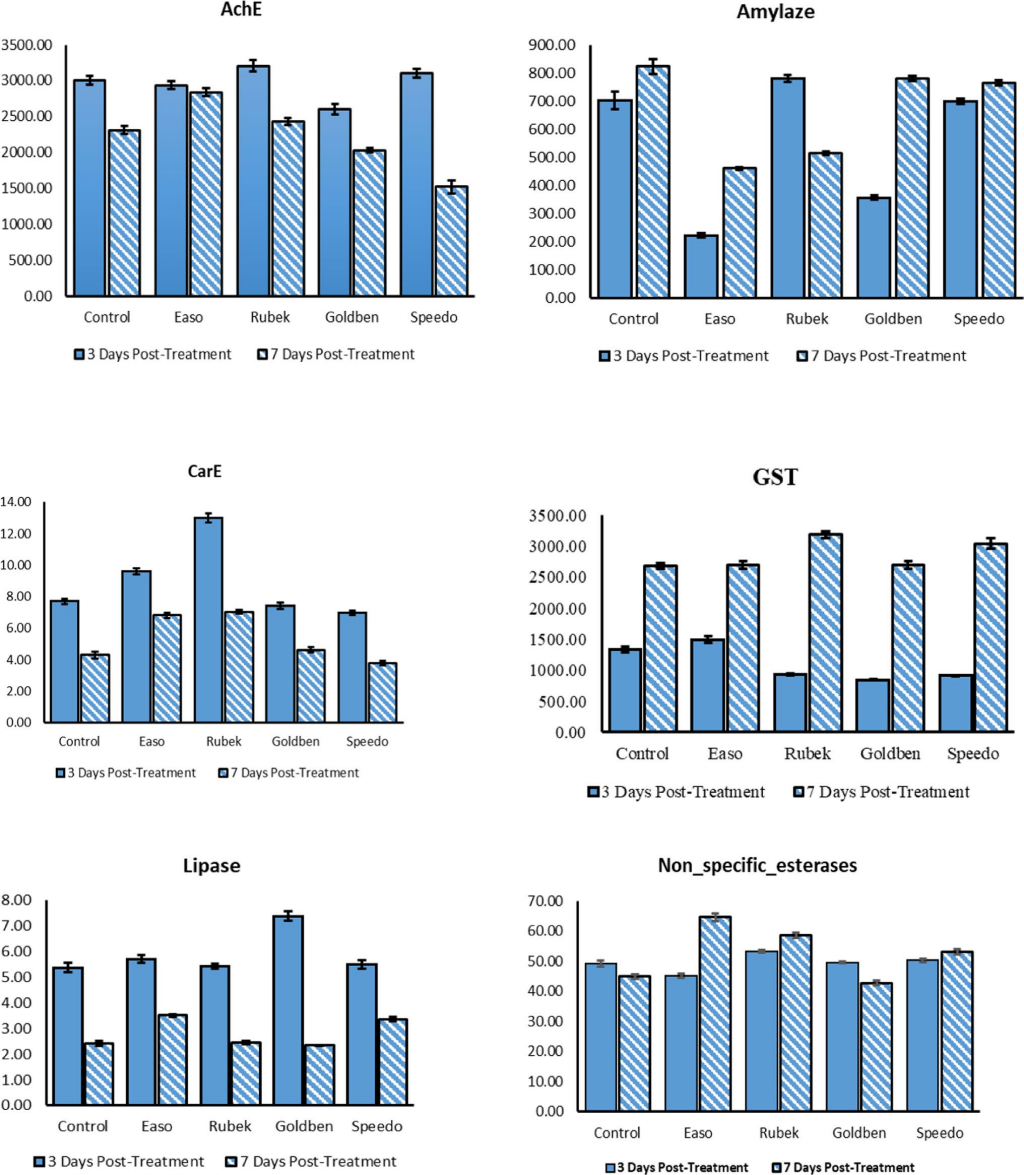

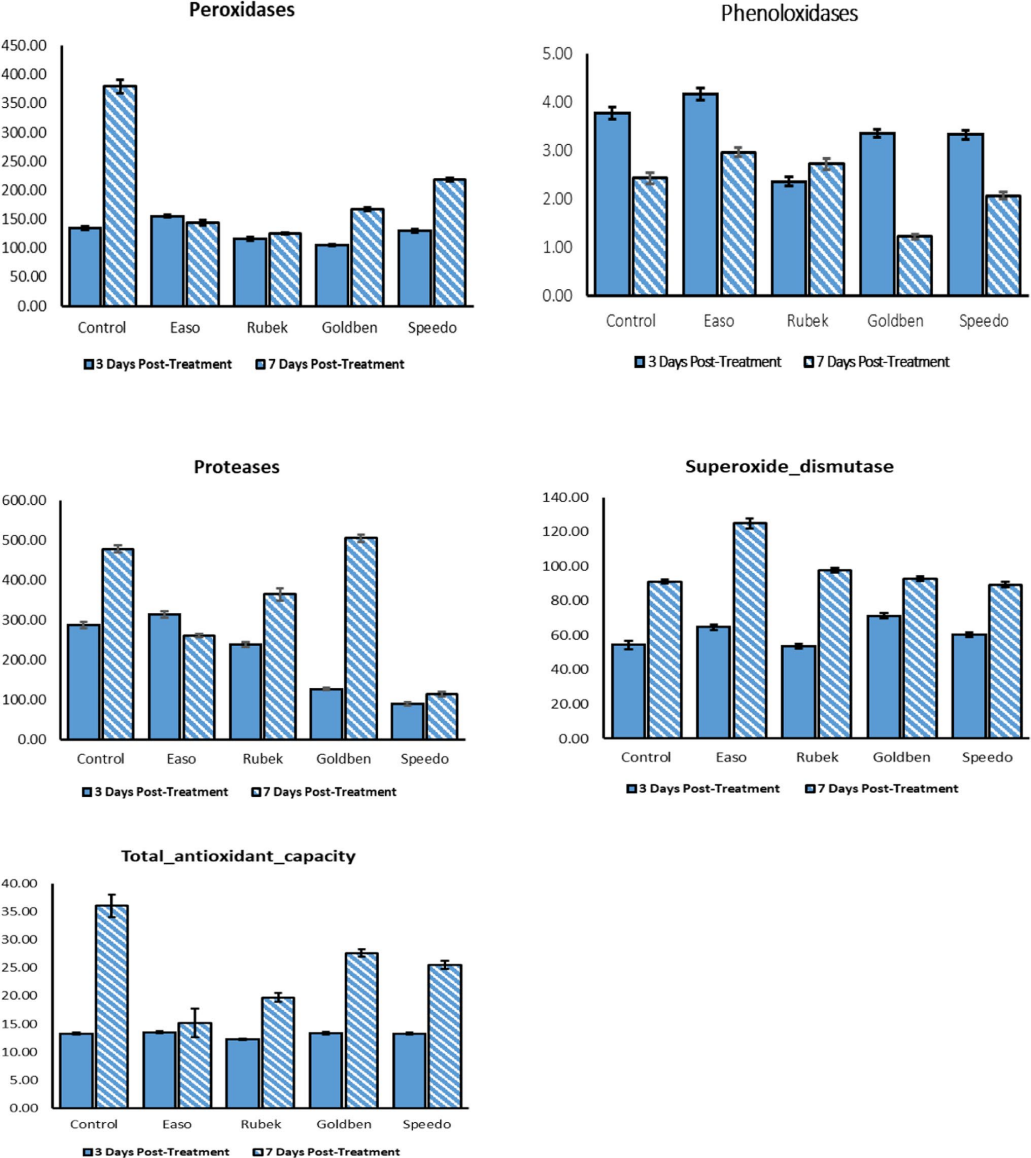
Metabolic crosstalk between neurotoxicity, detoxification and antioxidant defense enzymes in *S. frugiperda* following exposure to tested insecticides.

A significant variation in enzyme activities was observed among different insecticide treatments over time, reflecting distinct metabolic and detoxification responses in *S. frugiperda* larvae (Fig. 1). These differences highlight variations in detoxification capacity, oxidative stress response, and metabolic adaptation following insecticide exposure. Acetylcholinesterase (AChE) activity decreased significantly over time (*p* < 0.001). At 3 days post-treatment, Rubek-treated larvae exhibited the highest AChE activity (3203.33 ± 79.65 U/mg protein), whereas at 7 days, Easo treatment showed the highest activity (2838.33 ± 51.99 U/mg protein). Overall, AChE activity varied significantly among treatments (*p* < 0.001), with the lowest activity observed in Speedo-treated larvae (1523.33 ± 90.25 U/mg protein). The reduction in AChE activity in certain treatments suggests potential neurotoxic effects or adaptive responses to prolonged exposure.

Amylase activity also showed significant treatment-dependent differences (*p* < 0.001). The Control group exhibited the highest activity at 7 days post-treatment (824.33 ± 26.87 U/mg protein), while Easo-treated larvae had the lowest (461.33 ± 4.67 U/mg protein), indicating possible metabolic shifts in carbohydrate digestion and utilization. Carboxylesterase (CarE) activity decreased significantly from 3 to 7 days across all treatments (*p* < 0.001). Rubek-treated larvae displayed the highest activity (7.05 ± 0.13 U/mg protein), whereas Speedo-treated larvae had the lowest (3.78 ± 0.15 U/mg protein). This decline suggests alterations in detoxification pathways following insecticide exposure. Glutathione S-transferase (GST), a key detoxification enzyme, exhibited substantial variation among treatments (*p* < 0.001). The highest GST activity was observed in Rubek-treated larvae (3183.67 ± 52.35 U/mg protein), while Easo-treated larvae had the lowest activity (2695.00 ± 57.95 U/mg protein), reflecting an adaptive response to oxidative stress. Lipase activity was significantly reduced at 7 days compared to 3 days post-treatment (*p* < 0.001). The lowest lipase activities were recorded in Goldben (2.34 ± 0.02 U/mg protein) and Rubek (2.46 ± 0.07 U/mg protein), whereas Easo-treated larvae showed the highest activity (3.51 ± 0.05 U/mg protein). These changes indicate shifts in lipid metabolism in response to insecticide exposure.

Non-specific esterase activity varied significantly among treatments (*p* < 0.001), with the highest activity in Easo-treated larvae (64.53 ± 1.32 U/mg protein) and the lowest in Goldben (42.77 ± 0.72 U/mg protein), indicating adaptive changes in ester hydrolysis pathways. Peroxidase (POD) activity was highest in the Control group (379.67 ± 12.14 U/mg protein, *p* < 0.001), reflecting a strong oxidative stress response. Phenoloxidase (PO) activity was significantly reduced across all treatments, ranging from 2.97 ± 0.09 U/mg protein in Easo to 1.23 ± 0.05 U/mg protein in Goldben (*p* < 0.001). Protease activity increased significantly from 3 to 7 days post-treatment (*p* < 0.001), with the highest activity in Goldben-treated larvae (504.33 ± 9.60 U/mg protein), followed by the Control group (477.67 ± 9.06 U/mg protein), suggesting enhanced protein metabolism and degradation. Superoxide dismutase (SOD) activity peaked in Easo-treated larvae (125.00 ± 2.89 U/mg protein, *p* < 0.001), indicating an elevated defense against oxidative stress. Total antioxidant capacity (TAC) was highest in the Control group (36.03 ± 2.06 U/mg protein), significantly exceeding all insecticide treatments (*p* < 0.001). The enzymatic activity values for all treatments are provided in supplementary file tables S1-S3.

### Gene expression results by qRT-PCR

**Fig. 2:**
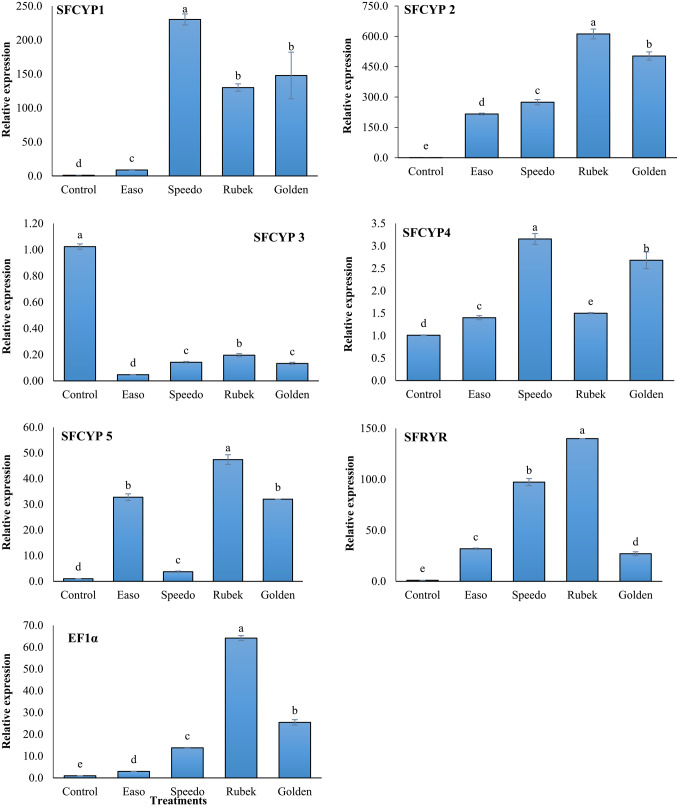
The impact of different insecticide application (Easo, Speedo, Rubek and Goldben) on relative expression level of ***S. frugiperda*** larvae genes (CYP1, CYP2, CYP3, CYP4, CYP5, RYR and EF1α at 7 days of treatment. Different letters on column indicate significant variations at 0.05.

As shown in Fig. 2, exposure to all tested insecticides induced significant changes in the expression of defense-related genes in *S. frugiperda* larvae at 7 days post-treatment. SFCYP1, SFCYP2, SFCYP4, SFCYP5, SFRYR, and EF1α were significantly upregulated compared to the control, with the most pronounced induction observed under Rubek treatment: SFCYP2 (612-fold), SFCYP5 (47.4-fold), SFRYR (140-fold), and EF1α (64.2-fold)

Application of Speedo led to increased expression of SFCYP1 and SFCYP4, with fold changes of 200- and threefold, respectively. In contrast, SFCYP3 was consistently downregulated across all insecticide treatments. The strongest downregulation was observed with Rubek (0.2-fold), while Easo induced the lowest SFCYP3 expression (0.05-fold) compared to the control.

Overall, Rubek exhibited the greatest effect on both up- and downregulated genes, highlighting its potent influence on detoxification and stress-related pathways.

### Genotoxicity assessment (Comet Assay)

**Fig. 3:**
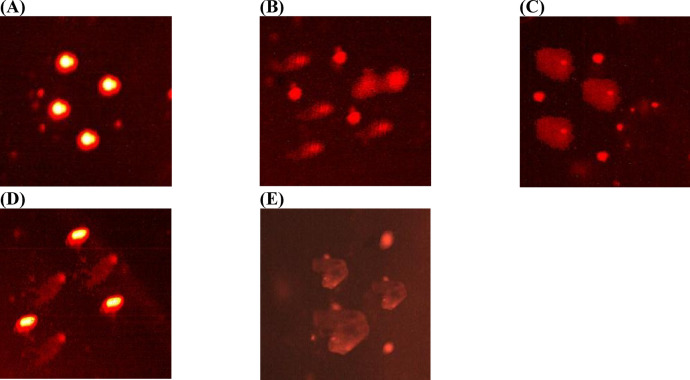
DNA damage in the 3rd-instar larvae of *S. frugiperda* exposed to different insecticides. (**A**) Control; (**B**) Larvae post-treatment with Indoxacarb; (**C**) Larvae treated with Methomyl; (**D**) Larvae treated with Acetamiprid + Bifenthrin mixture; (**E**) Larvae treated with Emamectin benzoate.

The comet assay (Fig. 3, Table 4) revealed distinct patterns of DNA damage in third-instar *S. frugiperda* larvae following exposure to the four tested insecticides. Compared with the control, all treatments induced significant increases (*p* < 0.05) in DNA fragmentation, as indicated by elevated tail length, % DNA in tail, and tail moment. Among the insecticides, emamectin benzoate caused the highest genotoxicity, with the greatest mean tail moment (1.97 ± 0.15) and % tail DNA (13.93 ± 0.33), reflecting substantial DNA strand breaks. Methomyl and the binary mixture of acetamiprid + bifenthrin induced moderate but significant DNA damage, with tail moments of 1.34 ± 0.09 and 1.26 ± 0.11, respectively, suggesting oxidative stress–related genotoxic effects. In contrast, indoxacarb-treated larvae exhibited comparatively lower DNA damage (tail moment 0.95 ± 0.04), despite its previously observed high resistance coefficient (RC = 12.86), implying that metabolic detoxification may mitigate direct DNA injury. Microscopic examination of hemocytes corroborated these findings. Control nuclei displayed compact, intact comets with minimal tail formation, whereas treated groups showed comets with elongated tails proportional to the extent of DNA damage. Notably, the pronounced DNA migration in emamectin-treated larvae was consistent with elevated oxidative stress markers and the upregulation of detoxification-related genes (SFCYP1, SFCYP2, and SFCYP4). Overall, the magnitude of DNA strand breaks followed the order: Emamectin benzoate > Acetamiprid + Bifenthrin > Methomyl > Indoxacarb > Control. These results indicate that sublethal exposure to the tested insecticides induces significant genotoxic stress in *S. frugiperda* larvae, with emamectin benzoate being the most potent. The observed genotoxic patterns align with biochemical and molecular responses, highlighting the roles of oxidative stress and cytochrome P450–mediated metabolism in resistance and toxicity mechanisms.

**Table 4:** Comet assay parameters showing DNA damage in third-instar larvae of *S. frugiperda* exposed to different insecticides.

Treatment group	Tail length (µm)	% DNA in tail	Tail moment
Control (untreated)	8.11 ± 0.47	8.50 ± 0.18	0.82 ± 0.03
Indoxacarb	7.58 ± 0.58	10.67 ± 0.23	0.95 ± 0.04
Methomyl	12.27 ± 0.63	11.78 ± 0.19	1.34 ± 0.09
Acetamiprid + Bifenthrin mixture	13.70 ± 0.59	12.29 ± 0.09	1.26 ± 0.11

### In silico analysis

#### Homology modelling

Protein receptor models were generated using homology-based 3D structure prediction in the MODELLER package. The resulting models were evaluated using several validation parameters. The Verify3D assessment showed a PASS, with averaged 3D–1D scores ≥ 0.1 for 85.32%, 84.6%, and 80.65% of residues in acetylcholine esterase, sodium channel protein, and glutamate-gated chloride channel receptors, respectively, confirming the reliability of the predicted structures. According to Table S4, residues fall into four Ramachandran plot regions: most favored, additionally allowed, generously allowed, and disallowed. For the three receptors in the same order, 89.5%, 82.8%, and 82.1% of residues were located in the most favored regions. An additional 10%, 14.8%, and 17.6% were found in the additionally allowed regions. Only 0.5%, 2.1%, and 0.3% of residues fell into the generously allowed regions. In the disallowed regions, merely 0.3% of residues were observed for the sodium channel protein, with none detected for the other receptors. Collectively, these validation parameters confirm the overall quality and reliability of the constructed protein models.

#### Molecular docking

**Fig. 4:**
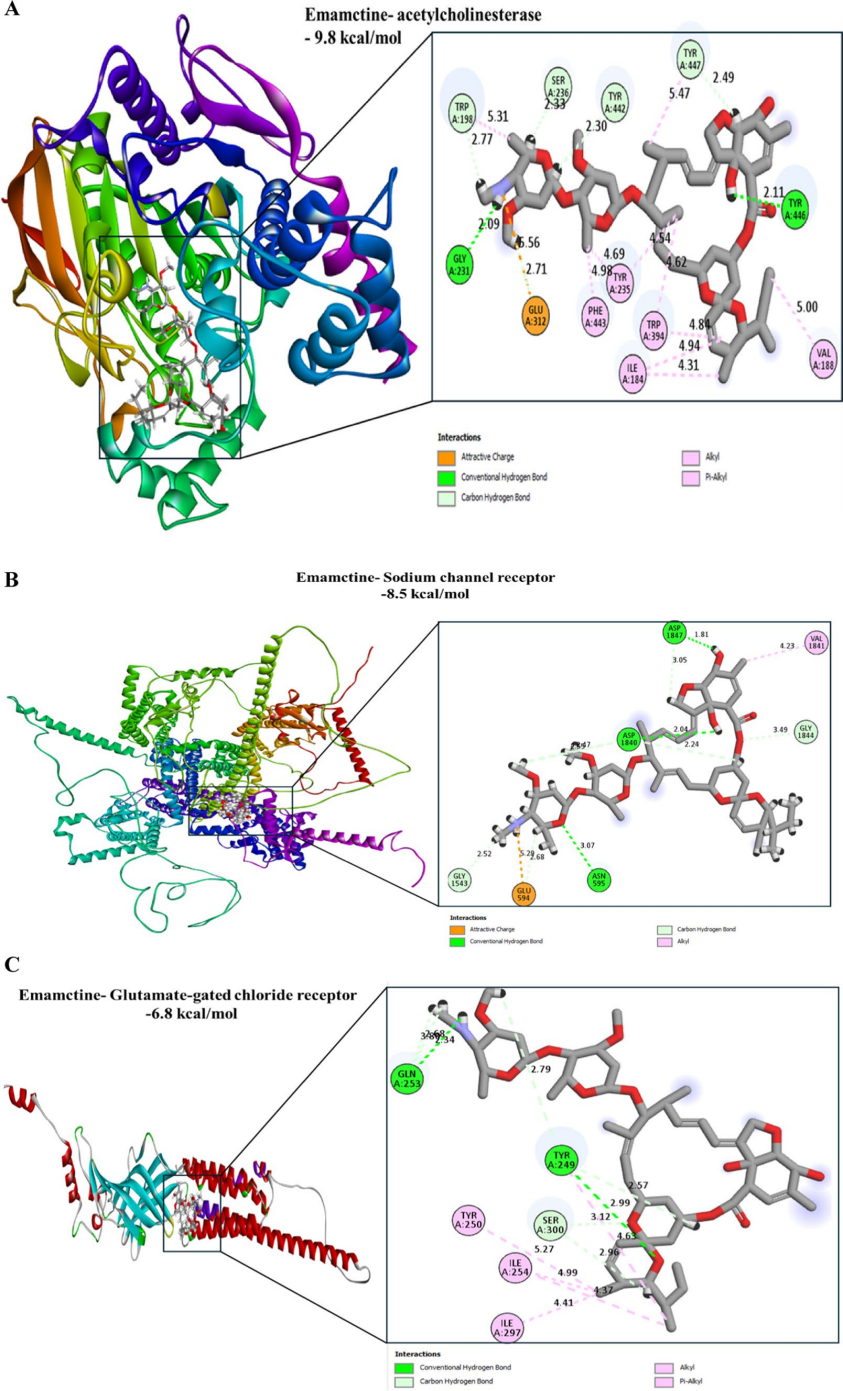
Docking interactions of Emamectin with active-site residues of target protein receptors; (**A**) acetylcholinesterase, (**B**) Sodium channel receptor, and (**C**) Glutamate-gated chloride receptor.

Based on Table S5 and Fig. 4, Emamectin exhibited strong binding affinity toward the key protein receptors involved in availability and development of *Spodoptera frugipedra*; -9.8 kcal/mol for acetylcholinesterase, -8.5 kcal/mol for sodium channel protein and -6.8 kcal/mol for glutamate-gated chloride channel receptors. The predicted interference and inhibition of these receptors by Emamectin are supported by its high molecular affinity, primarily driven by hydrophobic interactions formed with all protein receptors. In addition to hydrophobic contacts, hydrogen bonds were observed with specific residues: Gly231 and Trp446 in acetylcholinesterase; Asp1840, Asp1847, and Asn595 in the sodium channel receptor; and Gln253 and Tyr249 in the glutamate-gated chloride channel receptor. Collectively, these interactions contribute to the enhanced binding affinity of Emamectin toward the targeted protein receptors.

## Discussion

Understanding the susceptibility of *Spodoptera frugiperda* to insecticides is essential for effective pest management and for designing sustainable insecticide resistance management (IRM) strategies^47–49^. This study was conducted in Egypt, where *S. frugiperda* is a newly invasive pest and information on its insecticide resistance is scarce. We integrated bioassays, enzymatic analyses, molecular profiling, and genotoxicity assessments to evaluate short-term physiological and biochemical responses of third-instar larvae to insecticides with distinct modes of action (MOA). It is important to note that the present findings reflect immediate adaptive responses rather than confirmed multigenerational resistance evolution.

Our bioassay results revealed that emamectin benzoate was the most effective insecticide, showing the lowest LC50 with non-overlapping 95% confidence limits compared to other treatments. These variations likely reflect differences in MOA among the tested insecticides, and highlight emamectin benzoate’s potential role in IRM strategies^50,51^. The toxicity index (TI) further confirmed its superior efficacy over indoxacarb and methomyl, corroborating findings from Ahissou et al.^52^ and Shareef et al.^53^ using leaf-dipping or topical treatments. Emamectin benzoate is also considered eco-friendly, exhibiting rapid activity against *S. frugiperda* and other maize pests such as aphids and *Ostrinia* species, while negatively affecting larval biology and biochemistry^54–57^.

Indoxacarb and methomyl also displayed notable efficacy against the pest, consistent with reports by Kaiser et al.^58^**, **Ahissou et al.^52^**, **Thirawut et al.^57^**,** and Salem et al.^51^. The effectiveness of indoxacarb may relate to its multiple binding sites on the voltage-gated sodium channel (VGSC), enhancing its insecticidal activity^59^. Conversely, the binary mixture of acetamiprid and bifenthrin (1:1.2) was less effective. While individual applications of these compounds are often effective, their combination can lead to antagonistic or additive effects depending on pest strain and receptor subtypes^60–63^. The observed resistance of fourth-generation larvae to this mixture may reflect genetic background, target-site variability, or mixture ratio, aligning with reports from Benin and other regions^47,64^.

Importantly, larvae did not develop resistance to emamectin benzoate, indoxacarb, or methomyl, in agreement with previous studies^48,49,52,65^. Kaiser et al.^58^ further confirmed susceptibility of *S. frugiperda* to indoxacarb with no cross-resistance to other chemical groups, highlighting its potential in insecticide rotation programs. Gichere et al.^59^ emphasized that effective resistance management requires understanding insecticide binding sites, noting that indoxacarb’s three binding sites on VGSC increase its efficacy relative to pyrethroids with only one binding site.

Enzymatic analyses revealed significant metabolic adaptations over 7 days post-treatment. AchE and CarE activities decreased over time, suggesting a diminished neurotoxic response, while GST and peroxidase activities increased, reflecting enhanced detoxification and oxidative stress responses. Protease activity increased, indicating enhanced protein metabolism and potential stress-induced tissue remodeling^66^, whereas lipase activity declined, suggesting metabolic shifts from lipid to carbohydrate utilization^49,67^. SOD and peroxidase elevations in control larvae further support oxidative stress responses^68^. These time-dependent enzymatic changes demonstrate adaptive metabolic responses that may provide early tolerance rather than fixed resistance. The decline in AchE activity contrasts with reports of enhanced insect mortality from AchE inhibition^69^, but aligns with Pedersen et al.^70^, who suggested compensatory detoxification mechanisms reduce reliance on AchE-mediated neuroprotection.

Cytochrome P450 monooxygenases (P450s), encoded by CYP genes, are central to detoxification, hormone regulation, molting, and xenobiotic metabolism in *Spodoptera* species^71–73^. In this study, SFCYP1, SFCYP2, SFCYP4, and SFCYP5 were strongly upregulated following insecticide exposure, indicating activation of detoxification pathways, whereas SFCYP3 was downregulated, suggesting differential substrate specificity or regulatory trade-offs^74,75^. Similarly, the ryanodine receptor (RyR), a target for certain insecticides, was upregulated, implying that the tested insecticides do not directly target RyR, but both P450 and RyR genes can serve as transcript markers of insecticide response^76–78^.

The comet assay confirmed that sublethal insecticide exposure induced significant DNA strand breaks in hemocytes, with emamectin benzoate causing the greatest damage. Elevated tail DNA and tail moment values correlated with increased GST and peroxidase activities, suggesting ROS-mediated genotoxicity. These findings are consistent with reports in *Galleria mellonella*, *Apis mellifera*, *Helicoverpa armigera*, *Chironomus riparius*, and *Drosophila melanogaster*, where insecticide-induced ROS caused DNA fragmentation and apoptosis^79–85^.

Molecular docking results provided supportive computational evidence, showing that emamectin binds strongly to acetylcholinesterase, sodium channels, and glutamate-gated chloride channels.

Although Emamectin is primarily recognized as an allosteric modulator of the glutamate-gated chloride channel, our in silico docking results suggest that it may also exhibit relatively higher binding affinity toward acetylcholinesterase and sodium channel receptors compared with their corresponding reference insecticides, as indicated by lower predicted binding energies. These observations provide supportive computational insights that Emamectin could potentially interact with multiple receptor targets, which may partly contribute to its insecticidal activity. However, molecular docking simulations alone cannot confirm biological inhibition or receptor specificity, and therefore these results should be interpreted as predictive rather than definitive. Consequently, further biochemical and physiological studies are required to experimentally validate these interactions and clarify the underlying molecular mechanisms.

## Conclusion

This study provides an integrative understanding of the biochemical, molecular, and genotoxic responses of *Spodoptera frugiperda* to insecticide exposure. The observed elevation in GST and POD, along with modulation of key neuro-enzymes such as AChE, indicates dynamic adjustments in metabolic pathways as part of an acute stress response. The differential regulation of *CYP450* gene family members particularly the consistent upregulation of *SFCYP1*, *SFCYP2*, *SFCYP4*, *SFCYP5*, and *SFRYR* reflects an adaptive molecular defense system that enhances the insect’s resilience against xenobiotic stress. Moreover, the pronounced DNA damage detected demonstrates the genotoxic consequences of sublethal insecticide exposure, linking oxidative stress and DNA repair processes to early adaptive responses that may facilitate resistance development under prolonged exposure. These findings collectively reveal that *S. frugiperda* employs biochemical and molecular adjustments involving metabolic detoxification, oxidative stress modulation, and transcriptional changes under insecticide challenge**.** The integration of biochemical, molecular, and genotoxic biomarkers thus represents an integrative approach that may contribute to early detection of resistance when combined with further validation studies and for predicting the sustainability of control agents. Further experiments are required to confirm their predictive value for resistance development and to guide sustainable pest management strategies.

## Supplementary Information

Below is the link to the electronic supplementary material.

Supplementary Material 1

Supplementary Material 2

## Data Availability

The DNA sequence generated and analyzed during the current study has been deposited in the NCBI GenBank database. The sequence is publicly available under the accession number, PP658132.1.
