# Supplementary material for: Biochemical and molecular responses of Spodoptera frugiperda to insecticide exposure: detoxification enzymes, gene expression, and genotoxic effects
Source: Sci Rep. 2026 Apr 20;16:12887. doi: 10.1038/s41598-026-45372-w (PMC13096313; doi:10.1038/s41598-026-45372-w)
Supplement: Supplementary file 1 — Supplementary Material 1 [file 41598_2026_45372_MOESM1_ESM.docx]

**Table S1:** PROCHECK plot and statistics analysis of receptors models

| **protein** | **PROCHECK plot** | **PROCHECK statistics** | | | |
| --- | --- | --- | --- | --- | --- |
|  |  | **Most favored regions%** | **Additional Allowed regions%** | **Generously allowed regions%** | **Disallowed regions%** |
| **acetylcholine esterase** | 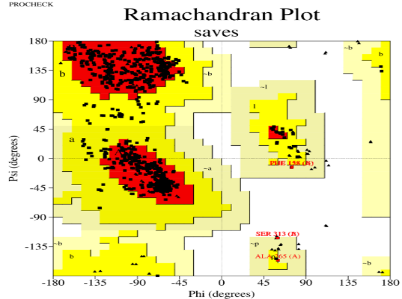 | **89.5%** | **10** | **0.5%** | **0.0** |
| **sodium channel protein** | 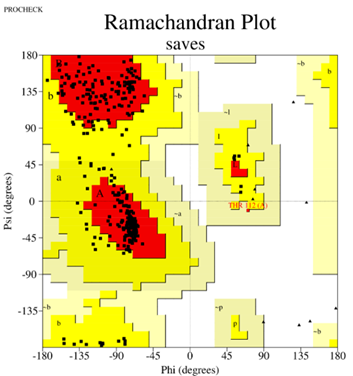 | **82.8%,** | **14.8** | **2.1%** | **0.3** |
| **Glutamate-gated chloride channel receptors** | 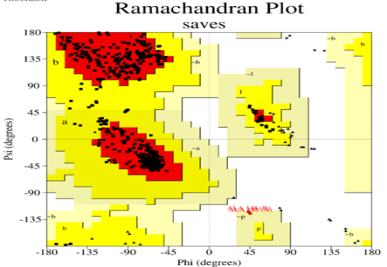 | **82.1%** | **17.6** | **0.3%** | **0.0** |

**Table S2:** Molecular interactions of Emamectin benzoate with the active-site residues of target protein receptor

| **Docking characters** | **acetylcholine esterase** | **sodium channel receptors** | **Glutamate-gated chloride channel receptors** |
| --- | --- | --- | --- |
| **interaction affinity score (kcal/mol), RMSD (Å)** | **- 9.8, 1.5** | **-8.5, 1.3** | **-6.8, 1.3** |
| **Interactions other than hydrogen bond** | **Alkyl, Pi-Alkyl, attractive charge, and caron hydrogen bond** | **Alkyl, attractive charge, and caron hydrogen** | **Alkyl, Pi-Alkyl, and caron hydrogen** |
| **Amino acids involved in hydrogen bonds: distance** | **Gly 231: 2.09**  **TRP 446: 2.11** | **ASP 1840: 2.04**  **ASP 1847: 1.8**  **ASN 595: 3.07** | **GLN 253: 2.3**  **TYR 249: 2.9** |
