# Supplementary material for: Biochemical and molecular responses of Spodoptera frugiperda to insecticide exposure: detoxification enzymes, gene expression, and genotoxic effects
Source: Sci Rep. 2026 Apr 20;16:12887. doi: 10.1038/s41598-026-45372-w (PMC13096313; doi:10.1038/s41598-026-45372-w)
Supplement: Supplementary file 2 — Supplementary Material 2 [file 41598_2026_45372_MOESM2_ESM.docx]

|  |  | ***Easo*** | | | ***Rubek*** | | | ***Goldben*** | | | ***Speedo*** | | | ***Control*** | | |
| --- | --- | --- | --- | --- | --- | --- | --- | --- | --- | --- | --- | --- | --- | --- | --- | --- |
| ***Enzyme*** |  | ***R_1_*** | ***R_2_*** | ***R_3_*** | ***R_1_*** | ***R_2_*** | ***R_3_*** | ***R_1_*** | ***R_2_*** | ***R_3_*** | ***R_1_*** | ***R_2_*** | ***R_3_*** | ***R_1_*** | ***R_2_*** | ***R_3_*** |
| ***Acetylcholinesterase (AchE)*** | ***3 days post-treatment*** | 2842 | 2951 | 3020 | 3360 | 3150 | 3100 | 2485 | 2600 | 2730 |  |  |  | 3116 | 3000 | 2905 |
|  | ***7 days post-treatment*** | 2880 | 2900 | 2735 | 2520 | 2400 | 2376 | 2100 | 2020 | 1981 | 1380 | 1500 | 1690 | 2300 | 2412 | 2225 |
| ***Amylase*** | ***3 days post-treatment*** | 212 | 222 | 235 | 777 | 805 | 765 | 360 | 369 | 341 |  |  |  | 760 | 700 | 653 |
|  | ***7 days post-treatment*** | 454 | 460 | 470 | 528 | 512 | 508 | 787 | 796 | 763 | 760 | 753 | 782 | 878 | 800 | 795 |
| ***Carboxylesterase***  ***(CarE)*** | ***3 days post-treatment*** | 9.32 | 9.50 | 10 | 12.5 | 13.5 | 13 | 7.2 | 7.3 | 7.8 |  |  |  | 8 | 7.6 | 7.5 |
|  | ***7 days post-treatment*** | 6.9 | 6.50 | 7.10 | 6.90 | 7.30 | 6.95 | 4.30 | 4.65 | 4.95 | 3.5 | 3.82 | 4.02 | 4.20 | 4 | 4.71 |
| ***Glutathione S-transferase***  ***(GST)*** | ***3 days post-treatment*** | 1607 | 1400 | 1485 | 952 | 930 | 915 | 840 | 855 | 833 |  |  |  | 1432 | 1300 | 1280 |
|  | ***7 days post-treatment*** | 2600 | 2685 | 2800 | 3280 | 3100 | 3171 | 2815 | 2609 | 2673 | 3200 | 3000 | 2915 | 2590 | 2700 | 2745 |
| ***Lipase*** | ***3 days post-treatment*** | 5.8 | 5.9 | 5.41 | 5.6 | 5.4 | 5.3 | 7.7 | 7.35 | 7.10 |  |  |  | 5 | 5.5 | 5.6 |
|  | ***7 days post-treatment*** | 3.60 | 3.49 | 3.44 | 2.40 | 2.39 | 2.60 | 2.30 | 2.36 | 2.35 | 3.40 | 3.20 | 3.51 | 2.50 | 2.20 | 2.55 |
| ***Non-specific esterases*** | ***3 days post-treatment*** | 43.6 | 45.1 | 46.5 | 52.5 | 53 | 54.2 | 49.4 | 50 | 49 |  |  |  | 49.1 | 51 | 47.5 |
|  | ***7 days post-treatment*** | 65.7 | 66 | 61.9 | 58.8 | 60 | 57.1 | 42.1 | 42 | 44.2 | 52.8 | 51.5 | 54.5 | 43.4 | 45 | 46.1 |
| ***Peroxidases*** | ***3 days post-treatment*** | 151 | 155 | 160 | 111 | 118 | 119 | 105 | 103 | 108 |  |  |  | 134 | 140 | 129 |
|  | ***7 days post-treatment*** | 135 | 145 | 152 | 123 | 125 | 129 | 172 | 170 | 160 | 216 | 215 | 225 | 358 | 400 | 381 |
| ***Phenoloxidase*** | ***3 days post-treatment*** | 4.1 | 4.40 | 4 | 2.30 | 2.25 | 2.55 | 3.20 | 3.35 | 3.50 |  |  |  | 4 | 3.70 | 3.60 |
|  | ***7 days post-treatment*** | 3.10 | 3 | 2.80 | 2.80 | 2.51 | 2.86 | 1.20 | 1.15 | 1.33 | 2.10 | 1.95 | 2.18 | 2.20 | 2.50 | 2.60 |
| ***Proteases*** | ***3 days post-treatment*** | 330 | 310 | 302 | 250 | 230 | 236 | 120 | 128 | 132 |  |  |  | 300 | 291 | 271 |
|  | ***7 days post-treatment*** | 252 | 261 | 269 | 396 | 350 | 345 | 502 | 489 | 522 | 100 | 120 | 122 | 490 | 483 | 460 |
| ***Superoxide dismutase***  ***( SOD)*** | ***3 days post-treatment*** | 62 | 64 | 68 | 52 | 56 | 53 | 71 | 74 | 69 |  |  |  | 50 | 55 | 58 |
|  | ***7 days post-treatment*** | 120 | 125 | 130 | 97 | 100 | 96 | 93 | 95 | 90 | 92 | 90 | 86 | 91 | 93 | 89 |
| ***Total antioxidant capacity*** | ***3 days post-treatment*** | 13.8 | 13.5 | 13.3 | 12.5 | 12.4 | 12 | 13.7 | 13.5 | 12.9 |  |  |  | 13.2 | 13.6 | 13 |
|  | ***7 days post-treatment*** | 18 | 17.5 | 10 | 20.9 | 20 | 18.3 | 28 | 28.5 | 26.4 | 24 | 26.2 | 26.3 | 35 | 40 | 33.1 |

**Supplementary table 1. Raw biochemical enzyme activity values (R1–R3 replicates) in *Spodoptera frugiperda* larvae following treatment with different insecticides at 3 and 7 days post-treatment.**

**Supplementary table 2. Effect of different insecticide on biochemical and antioxidant enzyme activities on *S. frugiperda* larvae 3 days post-treatment.**

| 3 days post-treatment | Easo | Rubek | Goldben | Control | *P-Value* |
| --- | --- | --- | --- | --- | --- |
| AChE | 2937.67±51.81^b^ | 3203.33±79.65^a^ | 2605.00±70.77^c^ | 3007.00±61.01^ab^ | 0.002 |
| amylase | 223.00 ±6.66^d^ | 782.33±11.85^a^ | 356.67±8.25^c^ | 704.33 ±30.96 ^b^ | <0.001 |
| CarE | 9.61±0.20^b^ | 13.00±0.29^a^ | 7.43±0.19^c^ | 7.70±0.15^c^ | <0.001 |
| GST | 1497.33±60.07^a^ | 932.33±10.74^c^ | 842.67±6.49^c^ | 1337.33±47.68^b^ | <0.001 |
| Lipase | 5.70±0.15^b^ | 5.43±0.09^b^ | 7.38±0.17^a^ | 5.37±0.19^b^ | <0.001 |
| Nonspecific esterase | 45.07±0.84^c^ | 53.23±0.50^a^ | 49.47±0.29^b^ | 49.20±1.01^b^ | <0.001 |
| Peroxidases | 155.33±2.60^a^ | 116.00±2.52^c^ | 105.33±1.45^d^ | 134.33±3.18^b^ | <0.001 |
| Phenol oxidases | 4.17±0.12^a^ | 2.37±0.09^d^ | 3.35±0.09^c^ | 3.77±0.12^b^ | <0.001 |
| Proteases | 314.00 ±8.33^a^ | 238.67±5.93^c^ | 126.67±3.53^d^ | 287.33 ±8.57^b^ | <0.001 |
| Superoxide dismutase | 64.67±1.76^b^ | 53.67±1.20^c^ | 71.33±1.45^a^ | 54.33±2.33^c^ | <0.001 |
| Total antioxidant capacity | 13.53±0.15^a^ | 12.30±0.15^b^ | 13.37±0.24^a^ | 13.27±0.18^a^ | 0.01 |

Values are expressed as **mean ± SE**. Means followed by different letters within the same row are **significantly different according to Duncan's multiple range test (p < 0.05)**. AChE: acetylcholinesterase; CarE: carboxylesterase; GST: glutathione S-transferase; SOD: superoxide dismutase.

| 7days post-treatment | Easo | Speedo | Rubek | Goldben | Control | *P-Value* |
| --- | --- | --- | --- | --- | --- | --- |
| AChE | 2838.33±51.99^a^ | 1523.33±90.25^d^ | 2432.00±44.54^b^ | 2033.67±35.03^c^ | 2312.33±54.33^b^ | <0.001 |
| amylase | 461.33±4.67^d^ | 765.00±8.74^b^ | 516.00±6.11^c^ | 782.00±9.85^ab^ | 824.33±26.87^a^ | <0.001 |
| CarE | 6.83±0.18^a^ | 3.78±0.15^c^ | 7.05±0.13^a^ | 4.63±0.19^b^ | 4.30±0.21^bc^ | <0.001 |
| GST | 2695.00±57.95^b^ | 3038.33±84.48^a^ | 3183.67±52.35^a^ | 2699.00±60.87^b^ | 2678.33±46.04^b^ | <0.001 |
| Lipase | 3.51±0.05^a^ | 3.37±0.09^a^ | 2.46±0.07^b^ | 2.34±0.02^b^ | 2.42±0.11^b^ | <0.001 |
| Nonspecific esterase | 64.53±1.32^a^ | 52.93±0.87^c^ | 58.63±0.84^b^ | 42.77±0.72^d^ | 44.83±0.78^d^ | <0.001 |
| Peroxidases | 144.00±4.93^d^ | 218.67±3.18^b^ | 125.67±1.76^d^ | 167.33±3.71^c^ | 379.67±12.14^a^ | <0.001 |
| Phenol oxidases | 2.97±0.09^a^ | 2.08±0.07^c^ | 2.72±0.11^a^ | 1.23±0.05^d^ | 2.43±0.12^b^ | <0.001 |
| Proteases | 260.67±4.91^c^ | 114.00±7.02^d^ | 363.67±16.23^b^ | 504.33±9.60^a^ | 477.67±9.06^a^ | <0.001 |
| Superoxide dismutase | 125.00±2.89^a^ | 89.33±1.76^c^ | 97.67±1.20^b^ | 92.67±1.45^bc^ | 91.00±1.15^c^ | <0.001 |
| Total antioxidant capacity | 15.17±2.59^c^ | 25.50±0.75^b^ | 19.73±0.76^c^ | 27.63±0.63^b^ | 36.03±2.06^a^ | <0.001 |

**Supplementary table 3. Effect of different insecticide on biochemical and antioxidant enzyme activities on *S. frugiperda* larvae 7 days post-treatment.**

Values are expressed as **mean ± SE**. Means followed by different letters within the same row are **significantly different according to Duncan's multiple range test (p < 0.05)**. AChE: acetylcholinesterase; CarE: carboxylesterase; GST: glutathione S-transferase; SOD: superoxide dismutase.

**Supplementary table 4. PROCHECK plot and statistics analysis of receptors models:**

| **protein** | **PROCHECK plot** | **PROCHECK statistics** | | | |
| --- | --- | --- | --- | --- | --- |
|  |  | **Most favored regions%** | **Additional Allowed regions%** | **Generously allowed regions%** | **Disallowed regions%** |
| **acetylcholine esterase** | 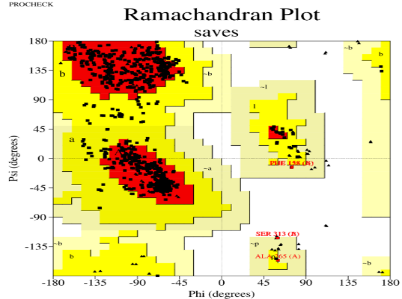 | **89.5%** | **10** | **0.5%** | **0.0** |
| **sodium channel protein** | 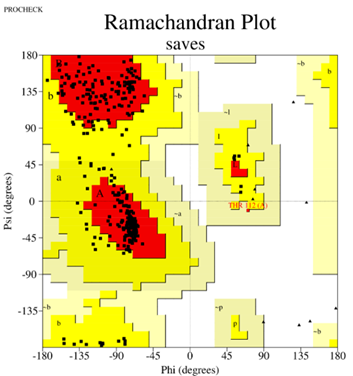 | **82.8%,** | **14.8** | **2.1%** | **0.3** |
| **Glutamate-gated chloride channel receptors** | 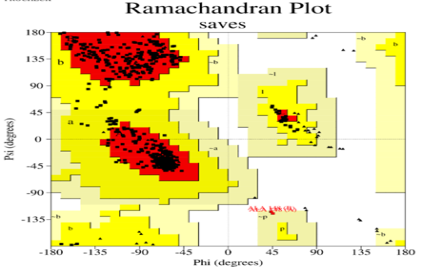 | **82.1%** | **17.6** | **0.3%** | **0.0** |

**Supplementary table 5. Molecular interactions of Emamectin benzoate with the active-site residues of target protein receptor**

| **Docking characters** | **acetylcholine esterase** | **sodium channel receptors** | **Glutamate-gated chloride channel receptors** |
| --- | --- | --- | --- |
| **interaction affinity score (kcal/mol), RMSD (Å)** | **- 9.8, 1.5** | **-8.5, 1.3** | **-6.8, 1.3** |
| **Interactions other than hydrogen bond** | **Alkyl, Pi-Alkyl, attractive charge, and caron hydrogen bond** | **Alkyl, attractive charge, and caron hydrogen** | **Alkyl, Pi-Alkyl, and caron hydrogen** |
| **Amino acids involved in hydrogen bonds: distance** | **Gly 231: 2.09**  **TRP 446: 2.11** | **ASP 1840: 2.04**  **ASP 1847: 1.8**  **ASN 595: 3.07** | **GLN 253: 2.3**  **TYR 249: 2.9** |
